# Soret and Dufour effects on unsteady MHD second-grade nanofluid flow across an exponentially stretching surface

**DOI:** 10.1038/s41598-022-16173-8

**Published:** 2022-07-12

**Authors:** Imran Siddique, Muhammad Nadeem, Jan Awrejcewicz, Witold Pawłowski

**Affiliations:** 1 Department of Mathematics, University of Management and Technology, Lahore, 54770 Pakistan; 2 Department of Automation, Biomechanics and Mechatronics, Lodz University of Technology, 1/15 Stefanowskiego St., 90-924 Lodz, Poland; 3 Institute of Machine Tools and Production Engineering, Lodz University of Technology, Lodz, Poland

**Keywords:** Mathematics and computing, Nanoscience and technology

## Abstract

The unsteady energy and mass transport of magnetohydrodynamics (MHD) second grade nanofluid via an exponentially extending surface with Dufour and Soret effects are investigated in this study. Variable thermal conductivity and mixed convection effects are used to investigate the heat transfer mechanism. There are also new characteristics such as slip flow, viscous dissipation, Brownian motion, nonlinear thermal radiation, and thermophoresis. In the problem formulation, the boundary-layer approximation is used. Using the suitable transformations, the energy, momentum, and concentration equations are generated into non-linear ordinary differential equations (ODEs). The solution to the resultant problems was calculated via the Homotopy analysis method (HAM). The effects of environmental parameters on velocity, temperature, and concentration profiles are graphically depicted. When comparing the current results to the previous literature, there was also a satisfactory level of agreement. In comparison to a flow based on constant characteristics, the flow with variable thermal conductivity is shown to be significantly different and realistic. The temperature of the fluid grew in direct proportion to the thermophoresis motion, buoyancy ratio, and Brownian motion parameters. According to the findings, the slippery porous surface may be employed efficiently in chemical and mechanical sectors that deal with a variety of very viscous flows.

## Introduction

Researchers have been interested in non-Newtonian materials since the last century because of their multidisciplinary nature and fascinating rheology. Non-Newtonian fluids are useful in a variety of industries, including processing, metals, chemical engineering, plastics, and food. Non-Newtonian liquids have a lot of useful applications such as glass blowing, biological fluids, cosmetics, artificial fibre, medicine, foodstuffs, metal spinning, shampoos etc. Non-Newtonian fluids also have a variety of properties and are divided into categories such as shear thickening, dilatant, shear-thinning, and thixotropic fluids. Rheologists have observed several fluid models, such as Maxwell, Casson, Williamson, Burgers, third grade, Oldroyd-B, micropolar, Jeffrey, Sutterby Cross, and Sisko, etc. Second-grade liquid, which describes shear thickening, shear-thinning, and Newtonian flow characteristics, however, behaves under certain conditions. Second-grade fluid is famous and worthy among researchers because of its dynamical characteristics^1–8^. On the other way, stretching a plastic sheet may not always be linear. For an exponentially stretched surface, the flow and heat transfer features can provide a greater variety of technical uses such as the heat instability at the expanding continuous surface, which increases exponentially with expanding velocity and temperature fluctuation, determines the result while annealing and thinning copper wires. Via Runge–Kutta (RK-4th) approach, The steady flow and heat transfer properties of a second-grade fluid with an exponentially extending surface were analyzed by Khan and Sanjayanand^9^. With the use of the Keller-box and HAM approach, Rehman et al.^10^ explored the steady flow of a second-grade fluid through an exponentially extending surface. Nadeem et al.^11^ scrutinized the heat and flow transmission of second-grade (viscoelastic) liquid across an exponential sheet. Ramzan and Bilal^12^ investigated unsteady, MHD, thermal radiation, and mixed convection flow of second-grade nanofluid caused by an extending sheet. Pakdemirli et al.^13^ scrutinized the characteristics of second-grade fluid using perturbation analysis. In recent years, there has been a lot of study on the second-grade liquid^14–23^.

Apart from the flow formed by an unsteady or steady extending/shrinking sheet, the impact of buoyancy force caused by the stretching sheets could not be ignored. Due to its vital applications in geothermal engineering, space technology, and nuclear cooling reactor, the topic of thermal radiation with mixed convective boundary layer (BL) flow has attracted a growing amount of attention. Engineers and scientists like an unsteady flow in many industrial systems because it gives them more control over their activities^24^. Also, the uninvited unsteady effect might develop around a system even in an ideal flow condition. Because of the additional time-dependent conditions in the equations, which distorted BL separation and the fluid flows pattern, the unstable BL flow behaviour is distinct from that of a steady flow^25^. Current design strategies that allow for enhanced system reliability, efficiency, and cost reduction of many dynamical types of equipment are attainable through a better knowledge of unsteady flow applications in engineering procedures^26^. Zaib et al.^27^ numerically described an unsteady flow with heat transport past an exponentially contracting surface.

Thermal radiation is the phenomenon of energy or heat transmission in the form of electromagnetic waves. The thermal radiation is significant in the high-temperature variance case among the ambient fluid and boundary surface. In engineering and physics, radiative impacts play an essential role. The effects of radiation heat transfer on various flows are essential in high-temperature and space technology processes. The effects of radiation are important in monitoring heat transmission in the polymer industries, where the final product quality is influenced by heat controlling variables to some extent. Also, the radiative impacts are significant in missiles, aircraft, gas turbines, solar radiations, space vehicles, liquid metal fluids, MHD accelerators, and nuclear power plants^24^. The impact of nonlinear thermal radiation on the Sakiadis flow was analysed by Pantokratoras and Fang^28^. The impact of thermal radiation and MHD flow of a water-based nanofluid in a shrinkable\stretchable divergent\convergent channel was examined by Dogonchi and Ganji^29^. The radiative flow via a porous surface was addressed by Khan et al.^30^. Numerous scholars^31–36^ are attracted the non-linear thermal radiation.

The Soret effect is associated with mass flux phenomena induced by heat diffusion, while the Dufour effect is related to the energy flux generated by the solute difference. The Soret impact is used to cope with concentrations of gases having lighter and medium molecular mass. Heat and mass transfer via the Soret and Dufour phenomena are significant in a wide range of industrial and engineering applications, like geosciences multicomponent melts, groundwater pollutant migration, solidification of binary alloys, chemical reactors, space cooling, isotope separation, oil reservoirs, and mixtures of gases^37^. Shojaei et al.^37^ used the second-grade fluid to examine the Dufour and Soret effect in a convective stretchy cylinder. In this work, they noticed that raising the viscoelastic parameter causes a rise in velocity profile, while the temperature and concentration profiles indicate the opposite behaviour. Similarly, The MHD nanofluid flow of two stretchable spinning discs was studied by Zangooee et al.^38^. The consequences of Soret and Dufour on MHD Casson fluid flow generated by a stretching sheet were inspected by Hayat et al.^39^. In the presence of heat-generating and nonlinear thermal radiation, Pal and Mondal^40^ numerically analyzed the thermophoretic, Soret, and Dufour impacts on steady-state flow via a non-isothermal wedge. Hayat et al.^41^ explored heat and mass transfer in a porous media filled with a viscoelastic fluid flow across a stretched vertical surface under Soret and Dufour effect. There are further investigations on the Dufour and Soret effects in Refs.^42–45^.

Standard heat transfer fluids will not be able to keep up with the required cooling efficiency. Because such fluids have lower heat conductivities, this is the case. The thermal conductivities of common heat transmission fluids including ethylene glycol, oil, and water are lower. Adding ultrafine nanoparticles to regular fluids can improve their thermal conductivities. As a result, nanofluids are thought to be the most effective way to boost the thermal conductivities of regular heat transfer fluids. Nanofluids are useful in a wide range of technological and industrial sectors, including heat exchangers, cooling electronic devices, automotive cooling, nuclear reactor cooling, transformer cooling, biomedicine, cancer tumour treatment and cancer therapy. Choi^46^ was the first to coin the term "nanofluid," and he demonstrated how adding nanoparticles to base liquids improves their thermal characteristics. After that, To examine the thermal performance of host fluids, Buongiorno^47^ established a scientific model. This model includes Brownian motion and thermophoresis. Hayat et al.^48^ used a convectively exponentially extending sheet to study MHD flow on second-grade nanofluid. Under a stretched sheet, Ahmad^49^ explored the unsteady flow of a second-grade nanofluid. The investigations provide further information on the nanofluid flow^50–60^.

The fluid that is impacted by boundary slip has a variety of uses, including cleaning artificial internal chambers and heart valves. At the micro-scale level, velocity is essential for liquid flow. Mustaffa et al.^61^ analyzed the performance of a slip-on nanofluid in a channel using HAM. Malvandietal^62^ used an analytical approach to study the combined effects of thermal slides and velocity slip on unsteady nanofluid stagnation point flow on a stretched surface. Khan^63^ explored the role of the isotropic squeezing flow of Cu-water and Cu-kerosene nanofluids on slip and viscous dissipation. Over an exponentially starching surface, Haider et al.^64^ employed a first-order slip-on viscous fluid. Also, the readers can learn about slip conditions^65–67^.

A comprehensive review of the previously listed literature, however, exposes some gaps. The authors have done their utmost to ensure that the information contained in this document is accurate and no previous studies have explored the unsteady slip and MHD nonlinear radiative flow of second-grade nanofluid caused by an exponentially permeable stretching sheet with variable thermal conductivity in their study outline. Heat and mass transport features are also investigated using viscous dissipation, Dufour, Soret, Brownian motion, and thermophoresis. The serious solutions of velocity, heat, and concentration fields are computed using the homotopy analysis technique (HAM). The skin friction and heat transfer rates are all influenced by many embedded flow factors, which are illustrated and described in depth through the graph.

## Mathematical model

**Figure 1:**
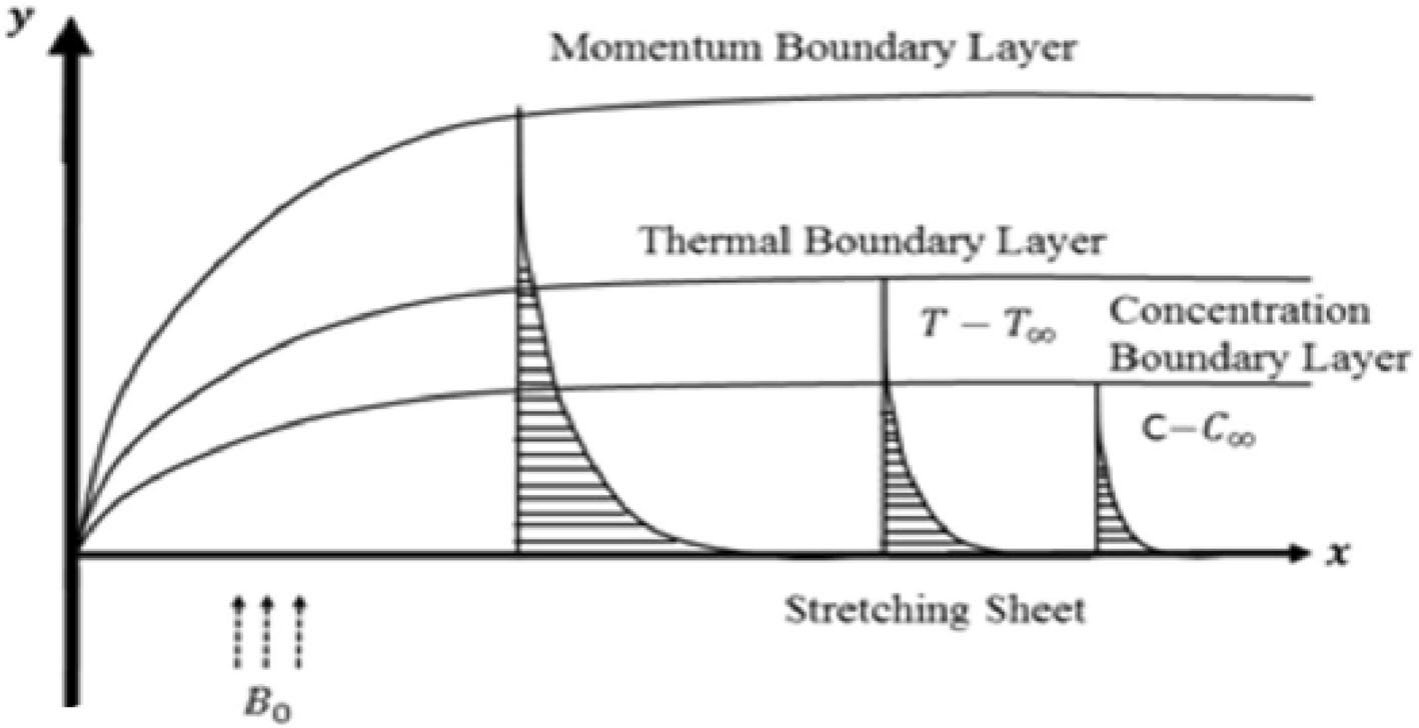
Flow geometry.

The time-dependent, 2D incompressible flow of MHD viscoelastic (Second-grade) nanofluid induced by an exponentially stretching surface is taken into account in this study, as exposed in Fig. 1. $u_{w} \left( {x,t} \right) = \left( {ae^{{{x \mathord{\left/ {\vphantom {x L}} \right. \kern-\nulldelimiterspace} L}}} /\left( {1 - ct} \right)} \right)$ represents the surface is stretched with the exponential velocity, where $v_{o}$ is constant, *L* indicates the characteristic length, and *c* denotes the unsteadiness. The ambient and reference temperatures and concentrations are designated as $T_{w} ,$ $T_{\infty } ,$ $C_{w} ,$ and $C_{\infty }$ respectively, whereas $T_{w} = T_{\infty } + e^{{{x \mathord{\left/ {\vphantom {x {2L}}} \right. \kern-\nulldelimiterspace} {2L}}}} \left( {T_{o} /\left( {1 - ct} \right)} \right)$ and $C_{w} = C_{\infty } + e^{{{x \mathord{\left/ {\vphantom {x {2L}}} \right. \kern-\nulldelimiterspace} {2L}}}} \left( {C_{o} /\left( {1 - ct} \right)} \right)$ determines the temperature and concentration distribution near the surface. The magnetic field is assumed to be $B\left( t \right) = \left( {B_{o} /\left( {1 - ct} \right)^{0.5} e^{{{x \mathord{\left/ {\vphantom {x {2L}}} \right. \kern-\nulldelimiterspace} {2L}}}} } \right),$ with $B_{o}$ denoting a uniform magnetic field.

The characteristics of the physical model and the mathematical model are considered under the following environments:

- Viscous dissipation.
- Variable thermal conductivity.
- Nonlinear radiative heat flux.
- Soret and dufour effects.
- Second-grade nanofluids.
- Impacts of Brownian and thermophoresis motion.
- Permeable sheet slippery edge constrain.

According to the following assumptions, the governing equations for continuity, momentum, heat and constration when using the BL approximation are^12,55–60,64^:

1 $$\frac{\partial v}{{\partial y}} + \frac{\partial u}{{\partial x}} = 0,$$

2 $$\begin{aligned} u\frac{\partial u}{{\partial x}} + \frac{\partial u}{{\partial t}} + v\frac{\partial u}{{\partial y}} & = \frac{{\mu_{f} }}{{\rho_{f} }}\frac{{\partial^{2} u}}{{\partial y^{2} }} + \frac{{\alpha_{1} }}{{\rho_{f} }}\left( {\frac{{\partial^{3} u}}{{\partial t\partial y^{3} }} + \frac{\partial u}{{\partial y}}\frac{{\partial^{2} v}}{{\partial y^{2} }} + u\frac{{\partial^{3} u}}{{\partial x\partial y^{3} }} + \frac{\partial u}{{\partial x}}\frac{{\partial^{2} u}}{{\partial y^{2} }} + v\frac{{\partial^{3} u}}{{\partial y^{3} }}} \right) - \frac{{\sigma_{f} }}{{\rho_{f} }}B_{0}^{2} u \\ & \quad + g\left[ {\left( {T - T_{\infty } } \right)\left( {\beta_{T} } \right)_{f} + \left( {C - C_{\infty } } \right)\left( {\beta_{C} } \right)_{f} } \right], \\ \end{aligned}$$

3 $$\begin{aligned} u\frac{\partial T}{{\partial x}} + v\frac{\partial T}{{\partial y}} + \frac{\partial T}{{\partial t}} & = \frac{1}{{\left( {\rho c_{P} } \right)_{f} }}\frac{\partial }{\partial y}\left( {K\left( T \right)\frac{\partial T}{{\partial y}}} \right) + \frac{{\mu_{f} }}{{\left( {\rho c_{P} } \right)_{f} }}\left( {\frac{\partial u}{{\partial y}}} \right)^{2} \\ & \quad + \frac{{\alpha_{1} }}{{\left( {\rho c_{P} } \right)_{f} }}\left( {\frac{\partial u}{{\partial y}}\frac{{\partial^{2} u}}{\partial y\partial t} + v\frac{\partial u}{{\partial y}}\frac{{\partial^{2} u}}{{\partial y^{2} }} + u\frac{\partial u}{{\partial y}}\frac{{\partial^{2} u}}{\partial x\partial y}} \right) \\ & \quad - \frac{{16\delta^{*} T_{\infty }^{3} }}{{3k^{*} \left( {\rho c_{P} } \right)_{f} }}\frac{{\partial^{2} T}}{{\partial y^{2} }} + \frac{{D_{T} k_{T} }}{{\left( {\rho c_{P} } \right)_{f} }}\frac{{\partial^{2} C}}{{\partial y^{2} }} + \frac{\tau }{{\left( {\rho c_{P} } \right)_{f} }}\left[ {D_{B} \frac{\partial T}{{\partial y}}\frac{\partial C}{{\partial y}} + \frac{{D_{T} }}{{T_{\infty } }}\left( {\frac{\partial T}{{\partial y}}} \right)^{2} } \right], \\ \end{aligned}$$

4 $$u\frac{\partial C}{{\partial x}} + v\frac{\partial C}{{\partial y}} + \frac{\partial C}{{\partial t}} = D_{B} \frac{{\partial^{2} C}}{{\partial y^{2} }} + \frac{{D_{T} k_{T} }}{{T_{\infty } }}\frac{{\partial^{2} T}}{{\partial y^{2} }},$$

the boundary conditions are:

5 $$\begin{aligned} & t < 0:\,T = T_{\infty } ,\,v = 0,\,u = 0,\,\forall \,x,y, \\ & t \ge 0:\,v = v_{w} ,\,u\left( {x,0} \right) = u_{w} + \mu_{f} \frac{\partial u}{{\partial y}},\,T = T_{w} \,{\text{at}}\,y \to 0, \\ & u = 0,\frac{\partial u}{{\partial y}} = 0,T = T_{\infty } \,{\text{at}}\,y \to \infty , \\ \end{aligned}$$

here $v$ and $u$ indicates the velocity component along the $y{\text{ - axis,}}$ and $x{\text{ - axis}}$ respectively, whereas the fluid temperature *T*. $\rho_{f}$ the density, gravity is *g*, The dynamic viscosity is $\mu_{f} ,$ $\delta_{f}$ the electrical conductivity, Stefan–Boltzmann constant $\delta_{f} ,$ mean absorption constant $k^{*} ,$ Brownian movement coefficient $D_{B} ,$ $\left( {\rho Cp} \right)_{f}$ is the heat capacity, magnetic field strength $B_{o}$ and $D_{T}$ thermophoresis diffusion coefficient.

In the temperature range of 0–400 F, thermal conductivity varies linearly. Variable thermal conductivity is therefore approximated as^53^:

$$K\left( T \right) = k_{*} \left( {1 - \delta_{*} \left( {T - T_{\infty } } \right)} \right),$$

where the empirical constants $\delta_{*}$, $K\left( T \right)$ variable thermal/heat conductivity, and $k_{*}$ coefficient of heat conductivity are far from the exponential plate. For fluids, $0 < \delta_{*} < 0$ can be positive, but for gases, it can be negative.

The following similarity transformations are illustrated for the governing Eqs. (1)–(4) with the constraints (5) in a much simple way^59^. Where the stream function $\omega$ can be specified as $v = - {{\partial \omega } \mathord{\left/ {\vphantom {{\partial \omega } {\partial x,\,{\text{and}}\,\,u = {{\partial \omega } \mathord{\left/ {\vphantom {{\partial \omega } {\partial y,}}} \right. \kern-\nulldelimiterspace} {\partial y,}}\,\,}}} \right. \kern-\nulldelimiterspace} {\partial x,\,{\text{and}}\,\,u = {{\partial \omega } \mathord{\left/ {\vphantom {{\partial \omega } {\partial y,}}} \right. \kern-\nulldelimiterspace} {\partial y,}}\,\,}}$ while the similarity variable is $\eta :$

6 $$\left. {\begin{array}{*{20}l} {\omega = \sqrt {\frac{{2la\nu _{f} }}{{1 - ct}}} e^{{x/2L}} f\left( \eta \right),\eta = \sqrt {\frac{a}{{2l\nu _{f} \left( {1 - ct} \right)}}} e^{{x/2L}} y,\theta \left( \eta \right) = \frac{{T - T_{\infty } }}{{T_{w} - T_{\infty } }},} \hfill \\ {u = \frac{{ae^{{x/L}} }}{{1 - ct}}f^{\prime } \left( \eta \right),v = - \sqrt {\frac{{a\nu _{f} }}{{2l\left( {1 - ct} \right)}}} e^{{x/2L}} \left( {\eta f^{\prime } \left( \eta \right) + f\left( \eta \right)} \right),\phi \left( \eta \right) = \frac{{C - C_{\infty } }}{{C_{w} - C_{\infty } }}.{\text{ }}} \hfill \\ \end{array} } \right\}$$

Equations (2)–(5) may be reduced to set of nonlinear ODEs in the setting of the above-mentioned relations by using the similarity transformations (6):

7 $$\begin{aligned} & f^{\prime\prime\prime} - \beta \left( {f^{\prime} + \frac{\eta }{2}f^{\prime\prime}} \right) - 2\left( {f^{\prime}} \right)^{2} + ff^{\prime\prime} - Mf^{\prime} + \alpha \left( {\beta f^{\prime\prime\prime} - \left( {f^{\prime\prime}} \right)^{2} - ff^{iv} + 2f^{\prime}f^{\prime\prime\prime} + \frac{\beta \eta }{2}f^{iv} } \right) \\ & \quad + Gr\theta + Gm\phi = 0, \\ \end{aligned}$$

8 $$\begin{aligned} & \left( {1 + \delta \theta + Nr\left( {1 + \theta \left( {\theta_{w} - 1} \right)} \right)^{3} } \right)\theta^{\prime\prime} + \delta \left( {\theta^{\prime}} \right)^{2} + 3Nr\left( {\theta^{\prime}} \right)^{2} \left( {\theta_{w} - 1} \right)\left( {1 + \theta \left( {\theta_{w} - 1} \right)} \right)^{2} - \Pr \theta f^{\prime} \\ & \quad + \Pr Df\phi^{\prime\prime} + \Pr f\theta^{\prime} + {\text{PrEc}} \left( {f^{\prime\prime}} \right)^{2} - \Pr \beta \left( {\frac{1}{2}\eta \theta^{\prime} + \theta } \right) \\ & \quad + \alpha {\text{PrEc}} f^{\prime\prime}\left( {2\eta f^{\prime\prime} + 2f^{\prime}f^{\prime\prime} + \eta \beta f^{\prime\prime\prime} - ff^{\prime\prime\prime}} \right) + \Pr \theta^{\prime}\left( {Nb\phi^{\prime} + Nt\theta^{\prime}} \right) = 0, \\ \end{aligned}$$

9 $$\phi^{\prime\prime} + Sc\left( {f\phi^{\prime} - \beta \phi - \frac{\beta \eta }{2}\phi^{\prime} - f^{\prime}\phi } \right) + ScSr\theta^{\prime\prime} = 0,$$

with the constraints

10 $$\begin{aligned} & f\left( \eta \right) = 0,f^{\prime}\left( \eta \right) = 1 + \gamma f^{\prime\prime}\left( \eta \right),\theta \left( \eta \right) = 1,\phi \left( \eta \right) = 1\quad {\text{at}}\quad \eta = 0, \\ & f^{\prime}\left( \eta \right) = 0,\theta \left( \eta \right) = 0,\phi \left( \eta \right) = 0\quad {\text{at}}\quad \eta \to \infty , \\ \end{aligned}$$

where the unsteadiness parameter is $\beta = 2Lc{\text{ }}/ae^{{x/L}} ,$ the magnetic parameter is $M = {{2\delta_{f} B_{o}^{2} \left( {1 - ct} \right)} \mathord{\left/ {\vphantom {{2\delta_{f} B_{o}^{2} \left( {1 - ct} \right)} {e^{{{x \mathord{\left/ {\vphantom {x L}} \right. \kern-\nulldelimiterspace} L}}} a\rho_{f} }}} \right. \kern-\nulldelimiterspace} {e^{{{x \mathord{\left/ {\vphantom {x L}} \right. \kern-\nulldelimiterspace} L}}} a\rho_{f} }},$ the Prandtl number is $\Pr = {{\nu_{f} } \mathord{\left/ {\vphantom {{\nu_{f} } {\alpha_{f} }}} \right. \kern-\nulldelimiterspace} {\alpha_{f} }},$ the second-grade fluid parameter is $\alpha = {{ae^{{{x \mathord{\left/ {\vphantom {x L}} \right. \kern-\nulldelimiterspace} L}}} } \mathord{\left/ {\vphantom {{ae^{{{x \mathord{\left/ {\vphantom {x L}} \right. \kern-\nulldelimiterspace} L}}} } {2L\mu_{f} \left( {1 - ct} \right)}}} \right. \kern-\nulldelimiterspace} {2L\mu_{f} \left( {1 - ct} \right)}},$ the Eckert number is $Ec = {{u_{w}^{2} } \mathord{\left/ {\vphantom {{u_{w}^{2} } {\left( {T_{w} - T_{\infty } } \right)\left( {C_{\rho } } \right)_{f} }}} \right. \kern-\nulldelimiterspace} {\left( {T_{w} - T_{\infty } } \right)\left( {C_{\rho } } \right)_{f} }},$ thermophoresis parameter is $Nt = {{\tau T_{o} D_{T} e^{{{x \mathord{\left/ {\vphantom {x {2L}}} \right. \kern-\nulldelimiterspace} {2L}}}} } \mathord{\left/ {\vphantom {{\tau T_{o} D_{T} e^{{{x \mathord{\left/ {\vphantom {x {2L}}} \right. \kern-\nulldelimiterspace} {2L}}}} } {\left( {\rho c} \right)_{f} \nu_{f} T_{\infty } }}} \right. \kern-\nulldelimiterspace} {\left( {\rho c} \right)_{f} \nu_{f} T_{\infty } }},$ Brownian motion parameter is $Nb = {{\tau T_{o}^{2} D_{B} \left( {\rho c} \right)_{\rho } e^{{{x \mathord{\left/ {\vphantom {x {2L}}} \right. \kern-\nulldelimiterspace} {2L}}}} } \mathord{\left/ {\vphantom {{\tau T_{o}^{2} D_{B} \left( {\rho c} \right)_{\rho } e^{{{x \mathord{\left/ {\vphantom {x {2L}}} \right. \kern-\nulldelimiterspace} {2L}}}} } {\nu \left( {\rho c} \right)_{f} }}} \right. \kern-\nulldelimiterspace} {\nu \left( {\rho c} \right)_{f} }},$ Schmidt number is $Sc = {{\nu_{f} } \mathord{\left/ {\vphantom {{\nu_{f} } {D_{B} }}} \right. \kern-\nulldelimiterspace} {D_{B} }},$ the slip parameter is $\gamma = a\sqrt {{{u_{w} e^{{{x \mathord{\left/ {\vphantom {x L}} \right. \kern-\nulldelimiterspace} L}}} } \mathord{\left/ {\vphantom {{u_{w} e^{{{x \mathord{\left/ {\vphantom {x L}} \right. \kern-\nulldelimiterspace} L}}} } {2L\nu_{f} \left( {1 - ct} \right)}}} \right. \kern-\nulldelimiterspace} {2L\nu_{f} \left( {1 - ct} \right)}}} ,$ the Dufour number is $Df = {{D_{T} K_{T} C_{o} } \mathord{\left/ {\vphantom {{D_{T} K_{T} C_{o} } {C_{s} \left( {cp} \right)_{f} \nu_{f} T_{o} ,}}} \right. \kern-\nulldelimiterspace} {C_{s} \left( {cp} \right)_{f} \nu_{f} T_{o} ,}}$ and the Soret number is $Sc = {{D_{T} K_{T} T_{o} } \mathord{\left/ {\vphantom {{D_{T} K_{T} T_{o} } {C_{o} \nu_{f} T_{\infty } .}}} \right. \kern-\nulldelimiterspace} {C_{o} \nu_{f} T_{\infty } }}$.

The coefficient of skin friction $\left( {{\text{C}}_{fx} } \right),$ the local Nusselt number $\left( {{\text{Nu}}_{{\text{x}}} } \right)$ and the local Sherwood-number $\left( {{\text{Sh}}_{{\text{x}}} } \right)$ are thus defined as:

11 $$C_{fx} = \frac{1}{{\rho_{f} u_{e}^{2} }}\left[ {\mu_{f} \frac{\partial u}{{\partial y}} + \alpha_{1} \left\{ {2\frac{\partial u}{{\partial y}}\frac{\partial u}{{\partial x}} + u\frac{{\partial^{2} u}}{\partial x\partial y} + v\frac{{\partial^{2} u}}{{\partial y^{2} }} + \frac{{\partial^{2} u}}{\partial t\partial y}} \right\}} \right]_{y = 0} ,$$

12 $$Nu_{x} = \frac{ - x}{{k_{f} \left( {T_{w} - T_{\infty } } \right)}}\left[ {\frac{{16\sigma^{*} T_{\infty }^{3} }}{{3k^{*} }}\frac{\partial u}{{\partial y}} + k_{f} \frac{\partial u}{{\partial y}}} \right]_{y = 0} ,$$

13 $$Sh_{x} = \frac{ - x}{{\left( {C_{w} - C_{\infty } } \right)}}\left( {\frac{\partial C}{{\partial y}}} \right)_{y = 0} .$$

Then we employ (6) into (11), (12) and (13), yielding the following relationship:

14 $$\sqrt {{\text{Re}}_{x} } C_{fx} = \left( {1 + \alpha \left( { - f^{\prime\prime}\left( 0 \right)f\left( 0 \right) + 7f^{\prime}\left( 0 \right)f^{\prime\prime}\left( 0 \right) + \eta f^{\prime\prime}\left( 0 \right)f^{\prime\prime}\left( 0 \right) + 3\beta f^{\prime\prime}\left( 0 \right) + \eta \beta f^{\prime\prime\prime}\left( 0 \right)} \right)} \right),$$

15 $$\left( {{\text{Re}}_{x} } \right)^{ - 0.5} Nu_{x} = - \left( {1 + Nr\left( {1 + \theta \left( 0 \right)\left( {\theta_{w} - 1} \right)} \right)^{3} } \right)\theta^{\prime}\left( 0 \right),$$

16 $$\left( {{\text{Re}}_{x} } \right)^{ - 0.5} Sh_{x} = - \phi^{\prime}\left( 0 \right),$$

where $Re_{x} = {{u_{e} x} \mathord{\left/ {\vphantom {{u_{e} x} {\nu_{f} }}} \right. \kern-\nulldelimiterspace} {\nu_{f} }}$ is the x-axis local Reynolds number.

### Solution by HAM

For solving highly nonlinear equations, the HAM is a powerful analytical tool. In light of boundary conditions 10, the HAM is used to resolve the generated Eqs. (7–10). The linear operators and Initial guesses are required to start the procedure using this method. As a result, we used $\left( {\Lambda_{f} ,\,\,\Lambda_{\theta } ,\,\,\Lambda_{\phi } } \right)$ as linear operators and $\left( {f_{0} \left( \eta \right),\,\,\theta_{0} \left( \eta \right),\,\,\phi_{0} \left( \eta \right)} \right)$ as initial estimations to solve momentum, energy and mass transform equations using the aforesaid method. For additional information on this technique, see^9–12,56–63^.

17 $$f_{0} \left( \eta \right) = \frac{1}{1 + \gamma }\left( {1 - e^{ - \eta } } \right),\quad \theta_{0} \left( \eta \right) = e^{ - \eta } ,\quad \phi_{0} \left( \eta \right) = e^{ - \eta } .$$

18 $$\Lambda_{f} \left[ {f\left( \eta \right)} \right] = f^{\prime\prime\prime} - f^{\prime},\quad \Lambda_{\theta } \left[ {\theta \left( \eta \right)} \right] = \theta^{\prime\prime} - \theta ,\quad \Lambda_{\theta } \left[ {\phi \left( \eta \right)} \right] = \phi^{\prime\prime} - \phi .$$

The properties of the aforesaid operator are as follows:

19 $$\left. \begin{gathered} \Lambda_{f} \left( {C_{1} + C_{2} e^{ - \eta } + C_{3} e^{\eta } } \right) = 0, \hfill \\ \Lambda_{\theta } \left( {C_{4} e^{ - \eta } + C_{5} e^{\eta } } \right) = 0, \hfill \\ \Lambda_{\phi } \left( {C_{6} e^{ - \eta } + C_{7} e^{\eta } } \right) = 0, \hfill \\ \end{gathered} \right\}$$

where $C_{js}$ ( j = 1, 2, …7) are arbitrary constants.

20 $$\left. \begin{gathered} \left( {1 - q} \right)\Lambda_{f} \left[ {\tilde{F}\left( {\eta ;q} \right) - \tilde{f}_{0} \left( \eta \right)} \right] - qh_{f} N_{f} \left[ {\tilde{F}\left( {\eta ;q} \right)} \right] = 0, \hfill \\ \left( {1 - q} \right)\Lambda_{\theta } \left[ {\tilde{\theta }\left( {\eta ;q} \right) - \tilde{\theta }_{0} \left( \eta \right)} \right] - qh_{\theta } N_{\theta } \left[ {\tilde{F}\left( {\eta ;q} \right),\,\tilde{\theta }\left( {\eta ;q} \right)} \right] = 0, \hfill \\ \Lambda_{\phi } \left[ {\tilde{\phi }\left( {\eta ;q} \right) - \tilde{\phi }_{0} \left( \eta \right)} \right] - qh_{\phi } N_{\phi } \left[ {\tilde{F}\left( {\eta ;q} \right),\,\tilde{\theta }\left( {\eta ;q} \right),\tilde{\phi }\left( {\eta ;q} \right)} \right] = 0, \hfill \\ \end{gathered} \right\}$$

where $h_{f} ,\,h_{\phi } \,\,{\text{and}}\,\,h_{\theta }$ signify non-zero auxiliary parameters and $q \in \left[ {0,\,1} \right]$ represents an embedding parameter while $\tilde{F},\,\,\tilde{\theta }\,\,{\text{and}}\,\,\tilde{\phi }$ representing the mapping occupations for $f,\,\,\theta \,\,{\text{and}}\,\,\phi .$

The boundary conditions become

21 $$\left. \begin{gathered} \tilde{F}\left( {0;q} \right) = 0,\,\,\tilde{F}^{\prime}\left( {0;q} \right) = 1 + \gamma \tilde{F}^{\prime\prime}\left( {0;q} \right),\,\,\,\tilde{\theta }\left( {0;q} \right) = 1,\,\,\tilde{\phi }\left( {0;q} \right) = 1, \hfill \\ \tilde{F}^{\prime}\left( {\infty ;q} \right) = 0,\,\,\,\tilde{\theta }\left( {\infty ;q} \right) = 0,\,\,\,\,\tilde{\phi }\left( {\infty ;q} \right) = 0. \hfill \\ \end{gathered} \right\}$$

22 $$\begin{aligned} N_{f} \left[ {\tilde{F}\left( {\eta ;q} \right)} \right] & = \frac{{d\tilde{F}^{\prime \prime \prime } \left( {\eta ;q} \right)}}{d\eta } - \beta \left( {\frac{{d\tilde{F}^{\prime } \left( {\eta ;q} \right)}}{d\eta } + \frac{\eta }{2}\frac{{d\tilde{F}^{\prime \prime } \left( {\eta ;q} \right)}}{d\eta }} \right) - 2\left( {\frac{{d\tilde{F}^{\prime } \left( {\eta ;q} \right)}}{d\eta }} \right)^{2} + \frac{{d\tilde{F}\left( {\eta ;q} \right)}}{d\eta }\frac{{d\tilde{F}^{\prime \prime } \left( {\eta ;q} \right)}}{d\eta } \\ & \quad + \alpha \left( {\beta \frac{{d\tilde{F}^{\prime \prime \prime } \left( {\eta ;q} \right)}}{d\eta } - \left( {\frac{{d\tilde{F}^{\prime \prime } \left( {\eta ;q} \right)}}{d\eta }} \right)^{2} - \tilde{F}\left( {\eta ;q} \right)\frac{{d\tilde{F}^{\prime \prime \prime \prime } \left( {\eta ;q} \right)}}{d\eta } + 2\frac{{d\tilde{F}^{\prime } \left( {\eta ;q} \right)}}{d\eta }\frac{{d\tilde{F}^{\prime \prime \prime } \left( {\eta ;q} \right)}}{d\eta } + \frac{\beta \eta }{2}\frac{{d\tilde{F}^{\prime \prime \prime \prime } \left( {\eta ;q} \right)}}{d\eta }} \right) \\ & \quad + Gr\tilde{\theta }\left( {\eta ;q} \right) + Gm\tilde{\phi }\left( {\eta ;q} \right) - M\frac{{d\tilde{F}^{\prime } \left( {\eta ;q} \right)}}{d\eta }, \\ \end{aligned}$$

23 $$\begin{aligned} N_{\theta } \left[ {\tilde{F}\left( {\eta ;q} \right),\tilde{\theta }\left( {\eta ;q} \right)} \right] & = \left( {1 + \delta \tilde{\theta }\left( {\eta ;q} \right) + Nr\left( {1 + \tilde{\theta }\left( {\eta ;q} \right)\left( {\theta_{w} - 1} \right)} \right)^{3} } \right)\frac{{d\tilde{\theta }^{\prime\prime}\left( {\eta ;q} \right)}}{d\eta } + \delta \left( {\frac{{d\tilde{\theta }^{\prime}\left( {\eta ;q} \right)}}{d\eta }} \right)^{2} \\ & \quad + 3Nr\left( {\frac{{d\tilde{\theta }^{\prime}\left( {\eta ;q} \right)}}{d\eta }} \right)^{2} \left( {\theta_{w} - 1} \right)\left( {1 + \tilde{\theta }\left( {\eta ;q} \right)\left( {\theta_{w} - 1} \right)} \right)^{2} - \Pr \tilde{\theta }\left( {\eta ;q} \right)\frac{{d\tilde{F}^{\prime}\left( {\eta ;q} \right)}}{d\eta } \\ & \quad + \Pr D\tilde{F}\left( {\eta ;q} \right)\frac{{d\tilde{\phi }^{\prime\prime}\left( {\eta ;q} \right)}}{d\eta } + \Pr \tilde{F}\left( {\eta ;q} \right)\frac{{d\tilde{\theta }^{\prime}\left( {\eta ;q} \right)}}{d\eta } + {\text{PrEc}} \left( {\frac{{d\tilde{F}^{\prime\prime}\left( {\eta ;q} \right)}}{d\eta }} \right)^{2} \\ & \quad - \Pr \beta \left( {\frac{1}{2}\eta \frac{{d\tilde{\theta }^{\prime}\left( {\eta ;q} \right)}}{d\eta } + \tilde{\theta }\left( {\eta ;q} \right)} \right) + \alpha {\text{PrEc}} \frac{{d\tilde{F}^{\prime\prime}\left( {\eta ;q} \right)}}{d\eta }\left( \begin{gathered} 2\eta \frac{{d\tilde{F}^{\prime\prime}\left( {\eta ;q} \right)}}{d\eta } + 2\frac{{d\tilde{F}^{\prime}\left( {\eta ;q} \right)}}{d\eta }\frac{{d\tilde{F}^{\prime\prime}\left( {\eta ;q} \right)}}{d\eta } \hfill \\ + \eta \beta \frac{{d\tilde{F}^{\prime\prime\prime}\left( {\eta ;q} \right)}}{d\eta } - \tilde{F}\left( {\eta ;q} \right)\frac{{d\tilde{F}^{\prime\prime\prime}\left( {\eta ;q} \right)}}{d\eta } \hfill \\ \end{gathered} \right) \\ & \quad + \Pr \frac{{d\tilde{\theta }^{\prime}\left( {\eta ;q} \right)}}{d\eta }\left( {Nb\frac{{d\tilde{\phi }^{\prime}\left( {\eta ;q} \right)}}{d\eta } + Nt\frac{{d\tilde{\theta }^{\prime}\left( {\eta ;q} \right)}}{d\eta }} \right), \\ \end{aligned}$$

24 $$\begin{aligned} N_{\phi } \left[ {\tilde{F}\left( {\eta ;q} \right),\tilde{\theta }\left( {\eta ;q} \right),\tilde{\phi }\left( {\eta ;q} \right)} \right] & = \frac{{d\tilde{\phi }^{\prime\prime}\left( {\eta ;q} \right)}}{d\eta } + Sc\left( \begin{gathered} \tilde{F}\left( {\eta ;q} \right)\frac{{d\tilde{\phi }^{\prime}\left( {\eta ;q} \right)}}{d\eta } - \frac{{d\tilde{F}^{\prime}\left( {\eta ;q} \right)}}{d\eta }\tilde{\phi }\left( {\eta ;q} \right) \hfill \\ - \beta \tilde{\phi }\left( {\eta ;q} \right) - \frac{\beta \eta }{2}\frac{{d\tilde{\phi }^{\prime}\left( {\eta ;q} \right)}}{d\eta } \hfill \\ \end{gathered} \right) \\ & \quad + ScSr\frac{{d\tilde{\theta }^{\prime\prime}\left( {\eta ;q} \right)}}{d\eta }, \\ \end{aligned}$$

The Eqs. (7)–(9) convert in the non-linear operators like Eqs. (22–24) then the series solution becomes:

25 $$\left. \begin{gathered} f\left( \eta \right) = f_{0} \left( \eta \right) + \sum\limits_{m = 1}^{\infty } {f_{m} \left( \eta \right)} , \hfill \\ \theta \left( \eta \right) = \theta_{0} \left( \eta \right) + \sum\limits_{m = 1}^{\infty } {\theta_{m} \left( \eta \right)} , \hfill \\ \phi \left( \eta \right) = \phi_{0} \left( \eta \right) + \sum\limits_{m = 1}^{\infty } {\phi_{m} \left( \eta \right)} , \hfill \\ \end{gathered} \right\}$$

## Results and discussion

On a second-grade nanofluid stream over an exponentially surface, an analysis of unsteady flow was performed. Due to the combination of viscous and nonlinear radiative heat, an electrically charged fluid is investigated in this scenario. An approximate analytical approach called HAM is used to handle the altered equations created from the said model. Examine the effects of dynamic parameters of the contours of our system on velocity, and temperature. Also, the comparison of variable and constant thermal conductivity through graphs. We have decided on the settings of key parameters for our simulation as *M* = 0.2, *Gr* = 0.2, *Gm* = 0.1, $$\beta = 0.3,$$$$\alpha = 0.4,$$ $$\sigma = 1.2,$$ *s* = 0.2, *Sc* = 0.2, *Sr* = 0.2, *Pr* = 1.2, *Nt* = 0.2, *Nb* = 0.2, *Df* = 0.1, $$\theta_{w} = 1.2,$$ *Nr* = 0.3, *Ec* = 0.3, and $$\gamma = 0.3.$$

To verify the numerical values of $\theta^{\prime}\left( 0 \right)$ with Haider et al.^64^, Table 1 was created. The numerical values acquired by the HAM in the current investigation were shown to be in great agreement with the literature.

**Table 1:** Comparison of present results of $\theta^{\prime}\left( 0 \right)$ with Haider et al.^64^ for variation in the *Pr* and *M* when H = 0.0, Nr = 0.0, $\beta = 0.0,$ Ec = 0.0.

M	Pr	Haider et al.^63^ (HAM)	Haider et al.^63^ (NM)	Present (HAM)
0	1	0.95478	0.95478	0.95477
	2	1.47146	1.47146	1.47145
	3	1.86907	1.86907	1.86906
	5	2.50012	2.50012	2.50012
	10	3.66027	3.66027	3.66026
1	1	0.56109	0.56109	0.56108

**Figure 2:**
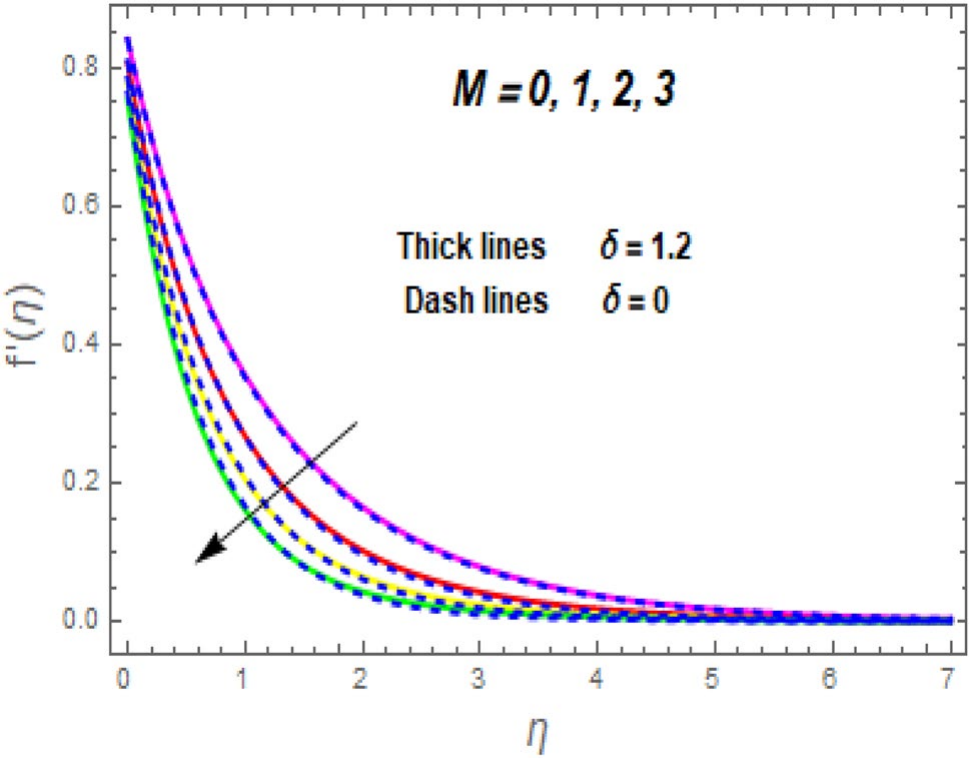
Variation of $f^{\prime}\left( \eta \right)$ for *M*.

**Figure 3:**
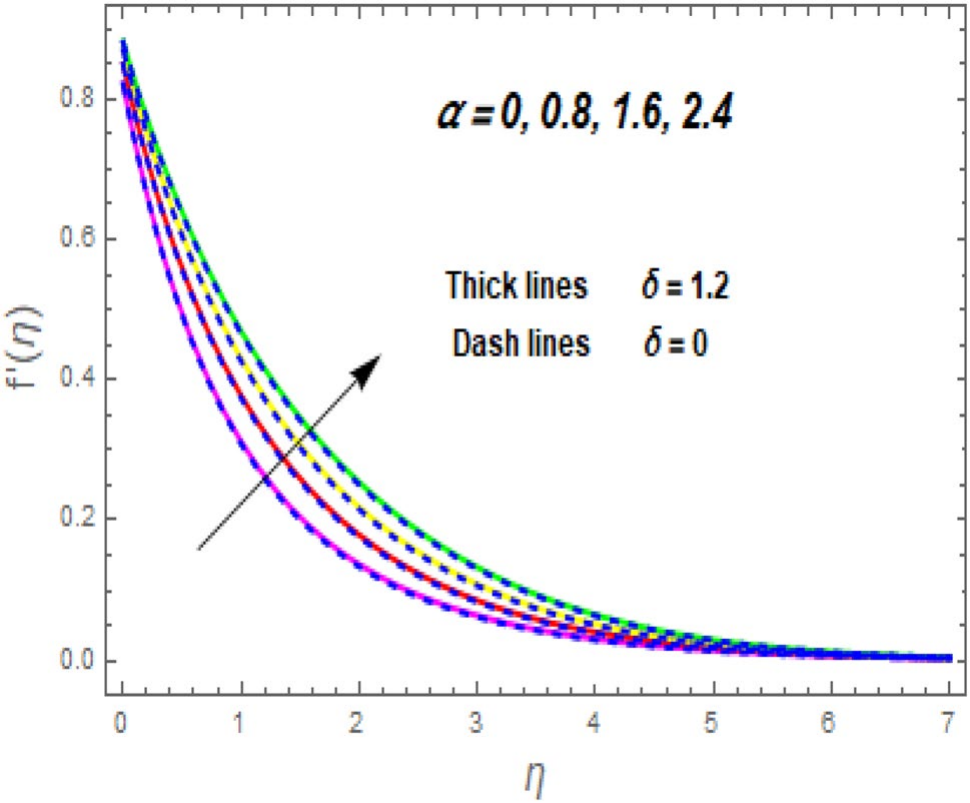
Variation of $f^{\prime}\left( \eta \right)$ for $\alpha .$

**Figure 4:**
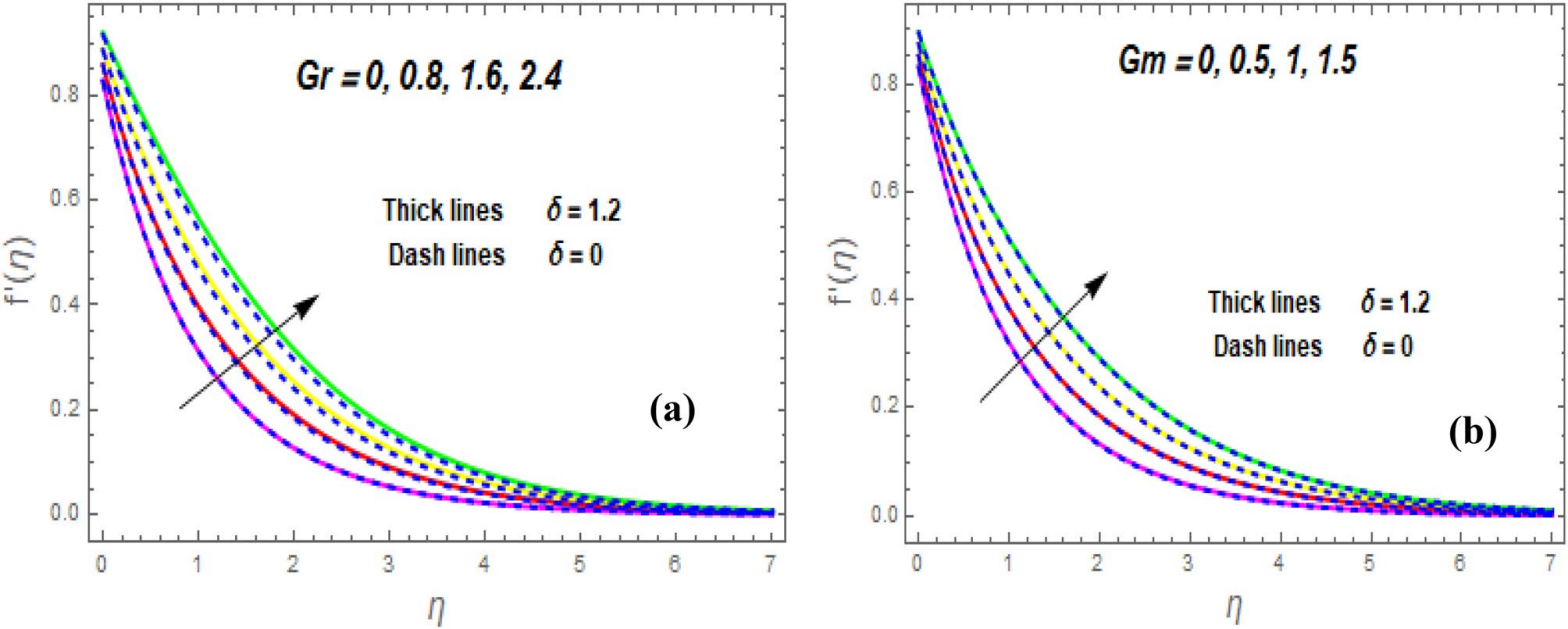
Variation of $f^{\prime}\left( \eta \right)$ for *Gr* (**a**) and *Gm* (**b**).

**Figure 5:**
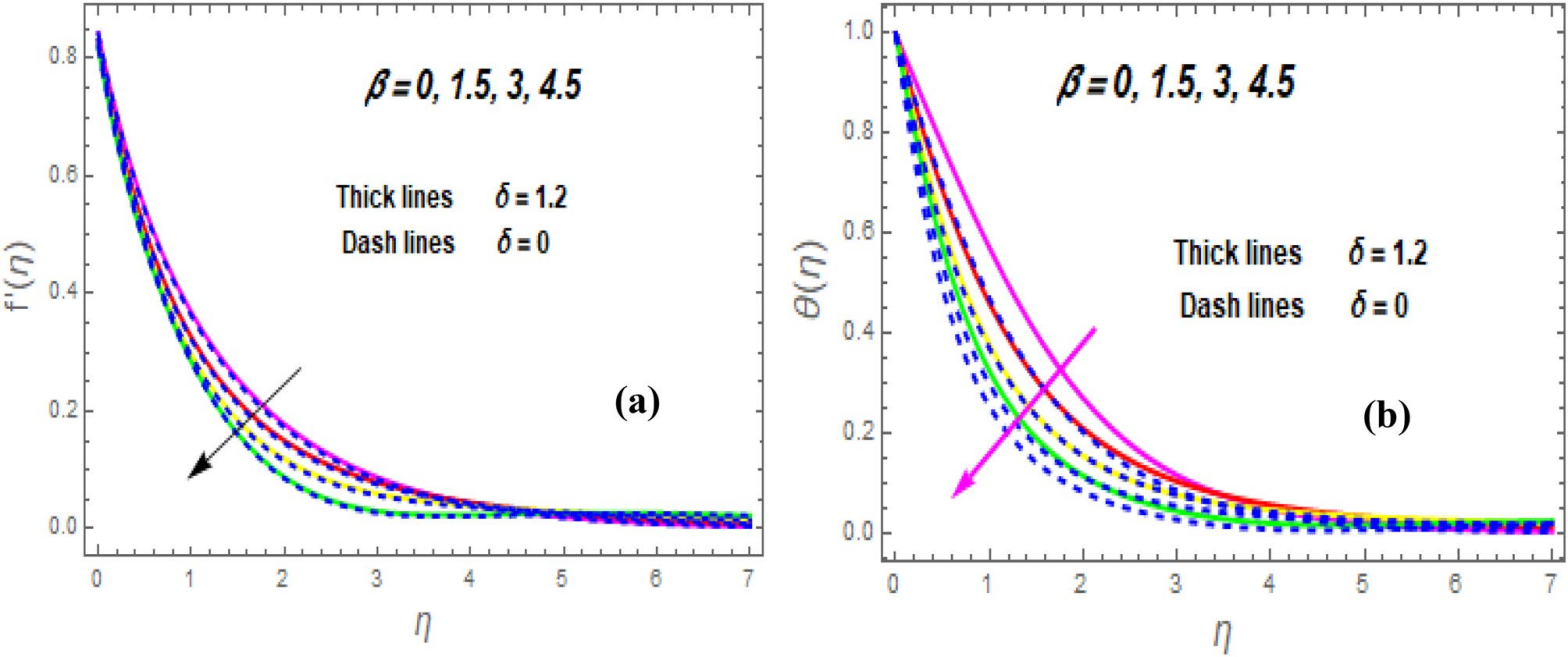
Variation of (**a**)$f^{\prime}\left( \eta \right)$ and (**b**) $\theta \left( \eta \right)$ for $\beta .$

**Figure 6:**
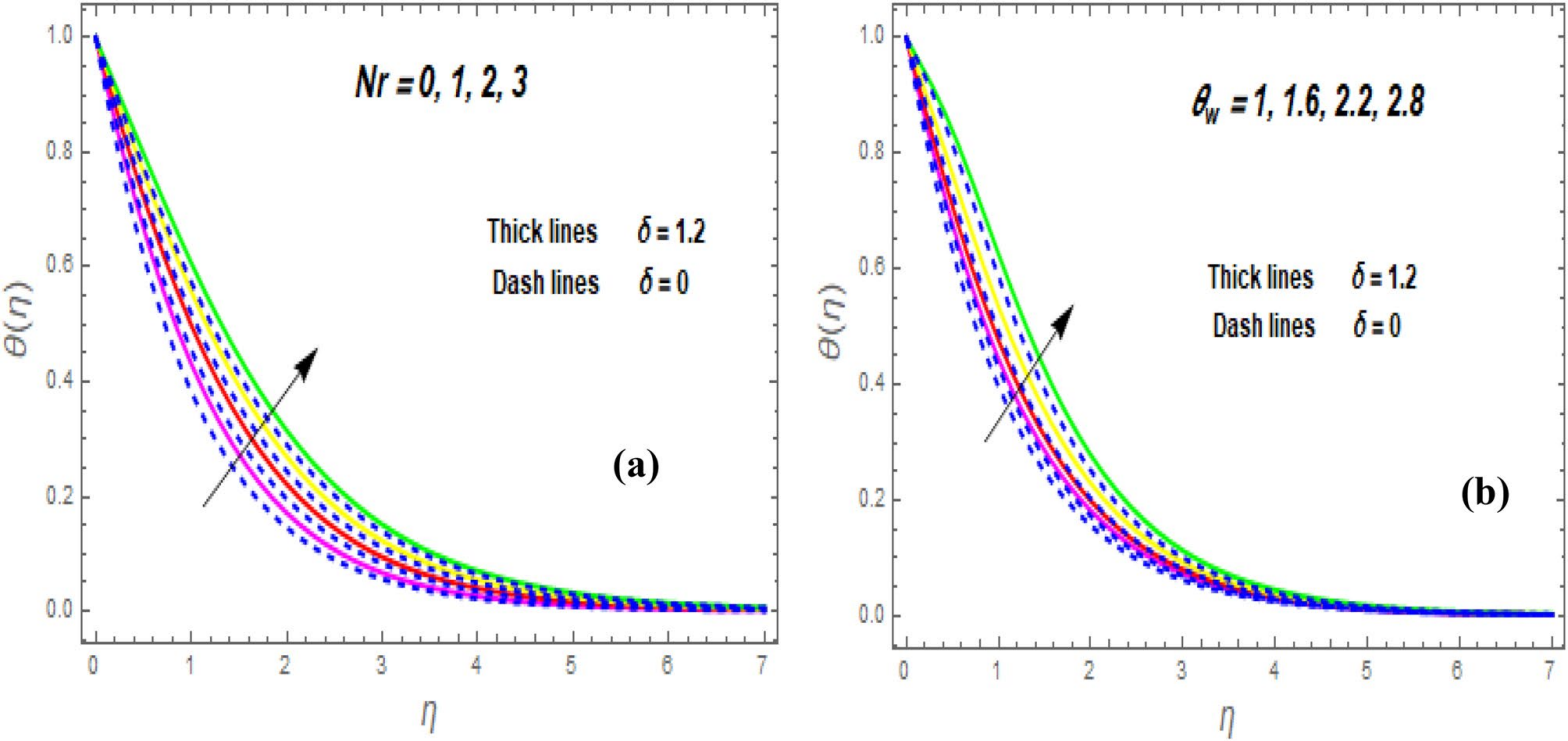
Variation of $\theta \left( \eta \right)$ for *Nr* (**a**) and $\theta w$ (**b**).

**Figure 7:**
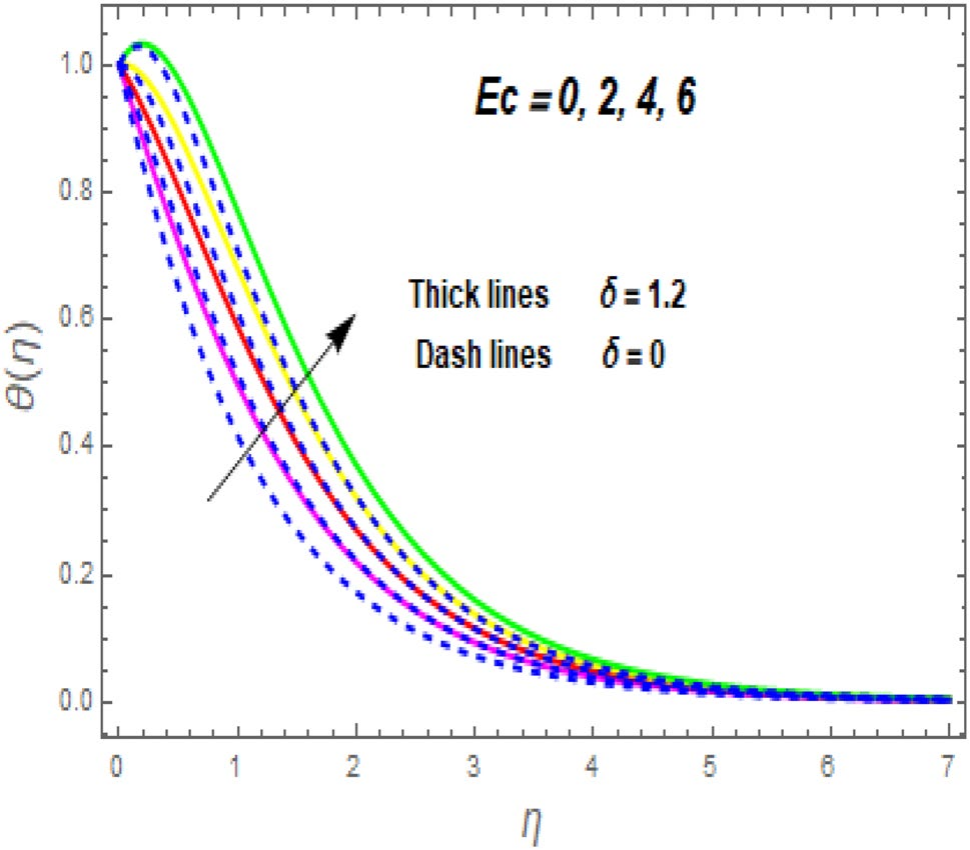
Variation of $\theta \left( \eta \right)$ for $Ec.$

**Figure 8:**
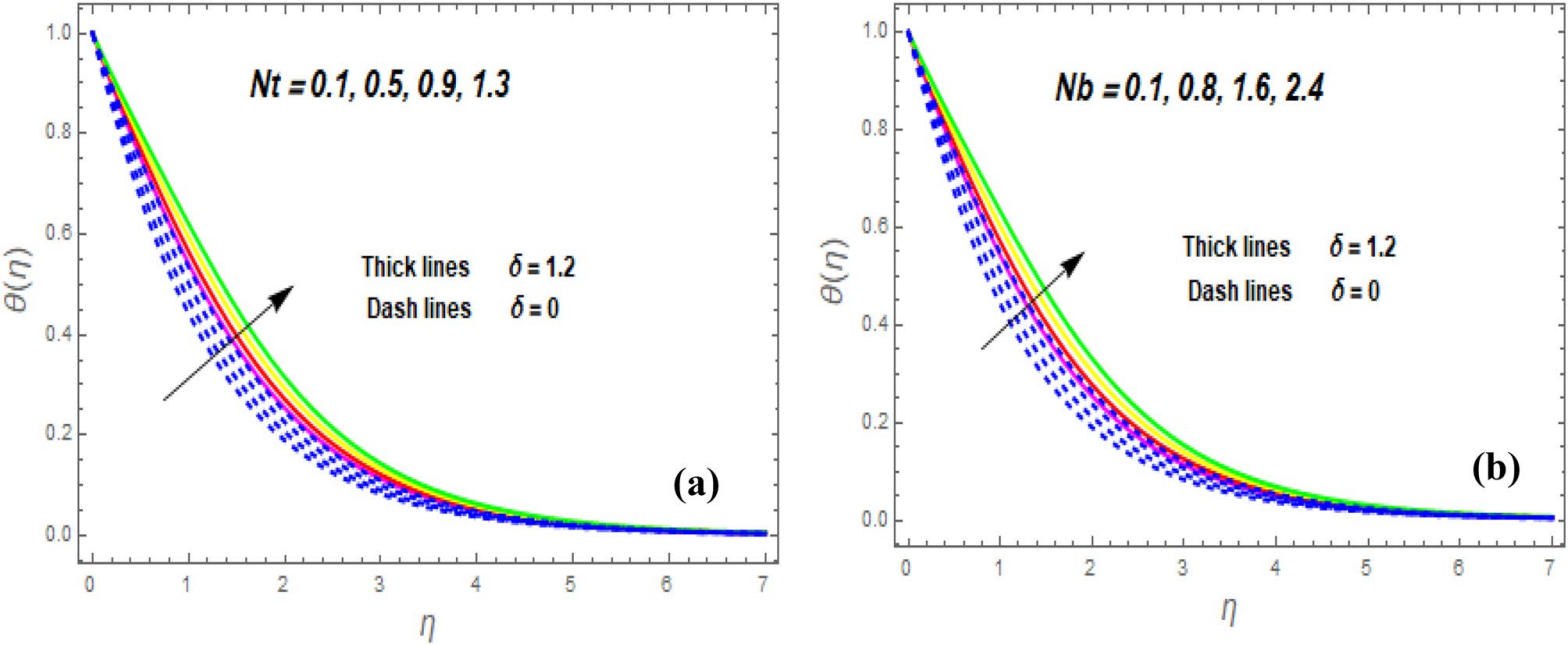
Variation of $\theta \left( \eta \right)$ for *Nt* (**a**) and *Nb* (**b**).

**Figure 9:**
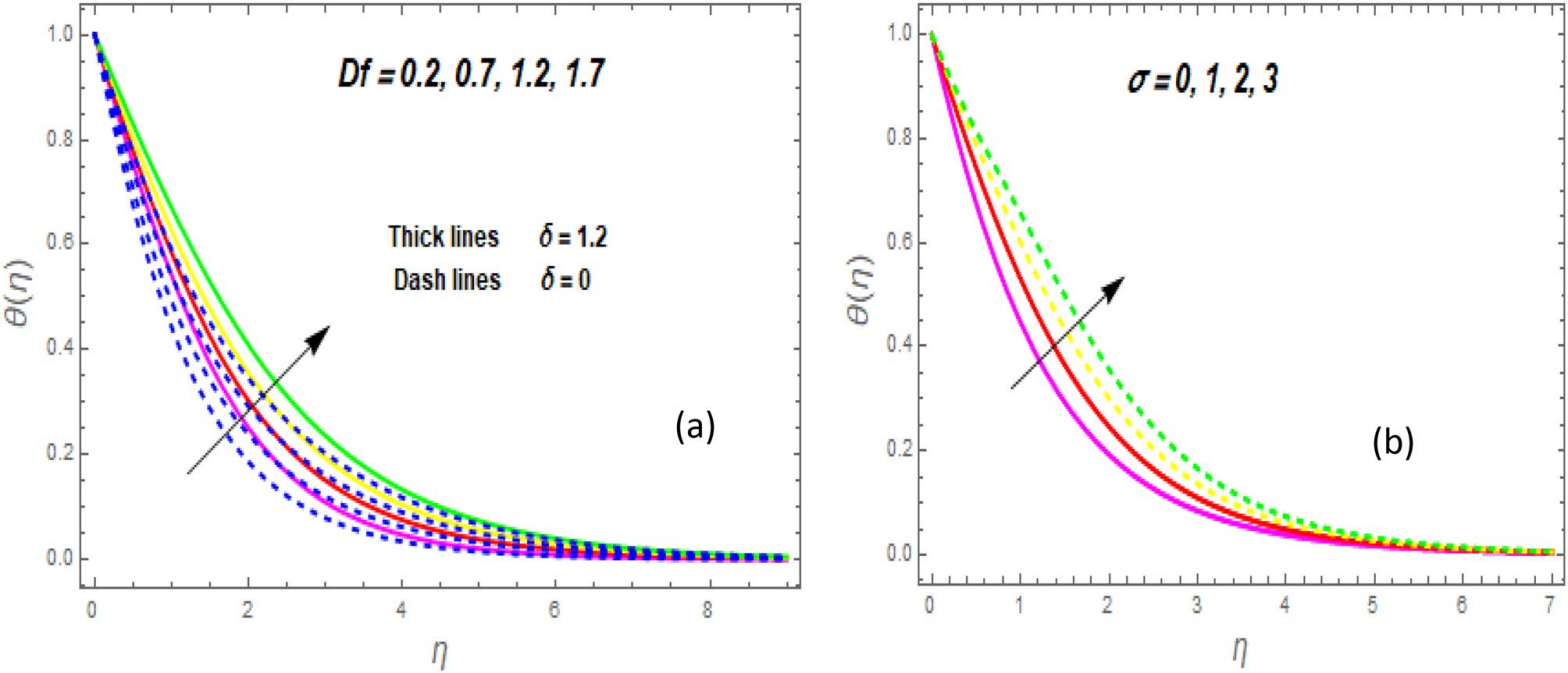
Variation of $\theta \left( \eta \right)$ for *D*f (**a**) and *δ* (**b**).

**Figure 10:**
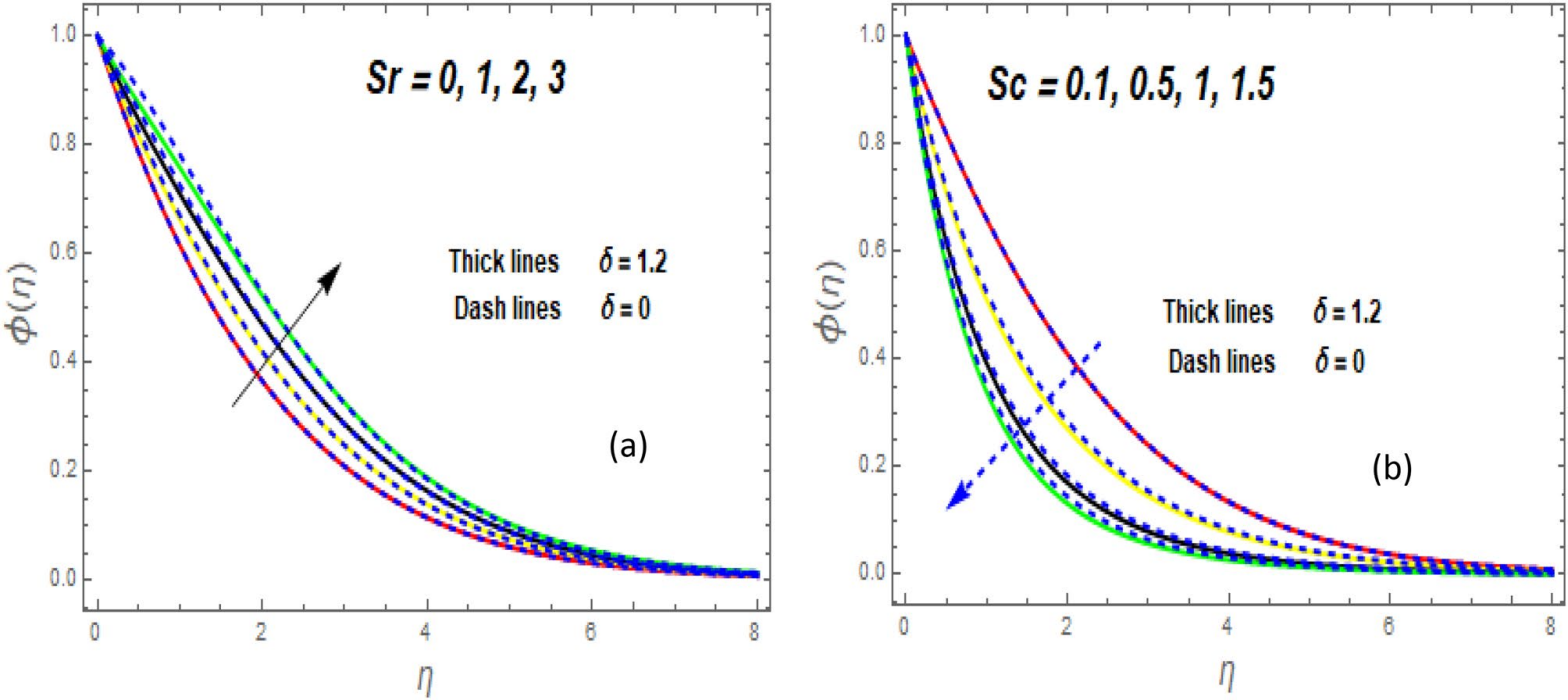
Variation of $\phi \left( \eta \right)$ for *Sr* (**a**) and *Sc* (**b**).

The effect of the magnetic parameter (M) on flow rate appears in Fig. 2. When *M* is elevated, the flow rate upsurges as well. Physically, magnetic strength provides resistive forces called Lorentz forces that oppose fluid flow, therefore, the fluid velocity drops. The deviation of the second-grade parameter ($\alpha$) on motion is portrayed in Fig. 3. It is revealed that a rise in $\alpha$ leads to an enhancement in velocity of both liquid and hybrid nanofluid. The reason is that, as alpha increases the viscous forces and fluid viscosity fall. Figure 4a,b demonstrates the effects of thermal Grashof (*Gr*) and mass or solutant Grashof (*Gm*) on flow rate. The amount of velocity was greatly influenced by an increase in the species or thermal buoyant force and hydromagnetic viscous force. The buoyancy and hydromagnetic viscous force, which tend to encourage fluid flow, are to blame. The fluid velocity is accelerated as a result. Figure 5 examines the influence of an unsteady component ($\beta$) on motion (a) and heat (b) profiles. With increasing $\beta ,$ the temperature and velocity profiles drop. This is because raising $\beta$ decreases the momentum and thermal BL. The relationship between the radiation parameter *Nr* and the temperature ratio parameter $\left( {\theta_{w} } \right)$ with the liquid temperature is seen in Fig. 6a,b. The random motion of particles is assisted by higher estimates of the *Nr*. Consequently, more particle collisions are observed, and more heat is created. So, there is a rise in the fluid heat. The thermal profile grows when the temperature ratio parameter $\left( {\theta_{w} } \right)$ upsurges, as perceived in Fig. 6b. These results are indicated that when $\theta_{w}$ grows, the temperature difference $\left( {T_{w} - T_{0} } \right)$ rises, causing the fluid temperature to rise. Figure 7 demonstrates that the behaviour of *Ec* is directly proportional to the temperature field, as *Ec* rises viscous dissipation upsurges, i.e., Heat energy is converted from kinetic energy, which raises the temperature. The inspiration of the thermophoresis parameter (*Nt*) on the heat flux is exposed in Fig. 8a. For larger values of *Nt*, the heat transfer rises. Thermophoresis is a transportation force that arises when a temperature differential exists between fluid layers. With a higher Thermophoresis value, the temperature differential between the layers grows, and the heat transformation rate grows as well. As a result, the temperature progressively rises, increasing the kinetic energy of nanoparticles. Figure 8b describes the inspiration of the Brownian motion parameter (*Nb*) on the heat flux. A rise in the *Nb* caused the temperature profile to settle at higher levels. *Nb* is the random motion caused by nanoparticles colliding with the base fluid. The *Nb* increases, and the collision rises. The internal kinetic energy of the fluid increases due to particle collisions. Figure 9a demonstrates the upshot of the Dufour (*Df*) on the temperature. Du denotes the caloric energy discharge during the flow being influenced by attention gradients. This is shown when the Du increases and the temperature distribution rises monotonically. The inspiration of the thermal conductivity parameter $\left( \delta \right)$ on heat flux is seen in Fig. 9b. The graph shows that when $\delta$ is amplified, the temperature escalations. Low thermal conductivity fluids have low temperatures, whereas high thermal conductivity fluids have high temperatures, as is well known. Larger thermal conductivity indicates that kinetic energy among molecules is more heightened, due to a great number of molecular collisions. The kinetic energy is transformed into thermal energy more quickly, resulting in more heat transfer. Figure 10a exhibitions the impact of Soret number (*Sr*) on the concentration profile. The temperature difference to the concentration quotient is denoted by *Sr*. The temperature gradient increases as *Sr* increases. The rate of molecular diffusion is thought to be increasing. As a result, for increasing *Sr* levels, the rate of mass transfer accelerates. Therefore, $\phi \left( \eta \right)$ improves. Figure 10b shows the upshot of the Schmidt number (*Sc*) on the particle concentration. With growing *Sc*, the concentration profile drop. The *Sc* is calculated by dividing the viscous rate of diffusion by the molecular rate of diffusion. Because of stabilising the molecular diffusion rate, the viscous diffusion rate increases, raising the *Sc.*

**Figure 11:**
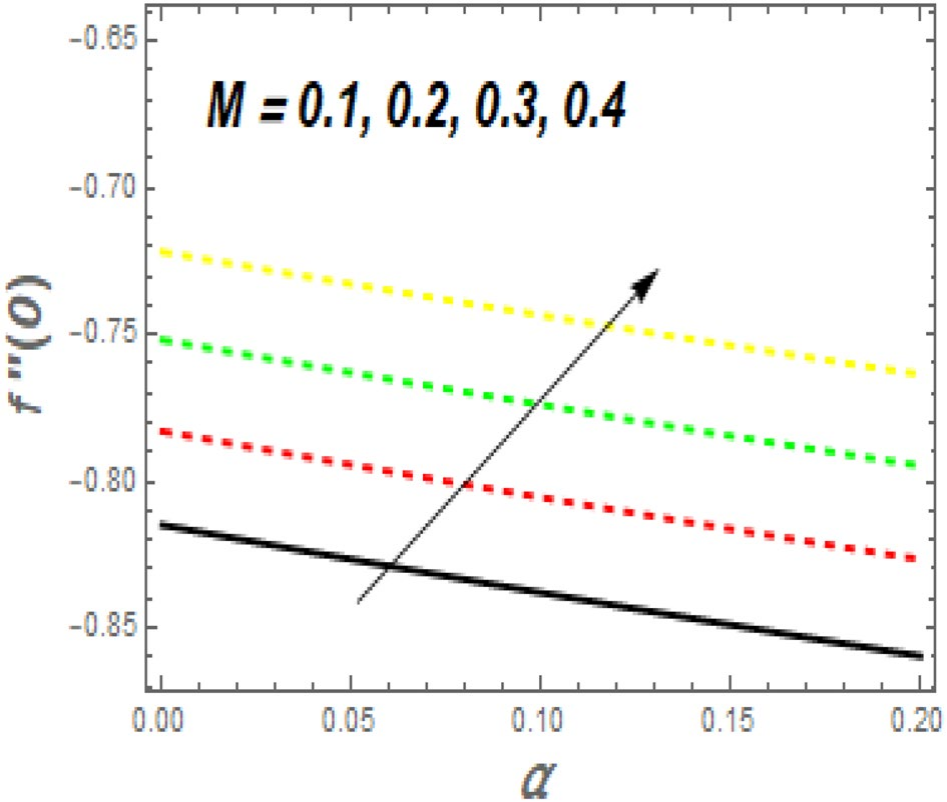
Impacts of *M* and $\alpha$ on $C_{f} .$

**Figure 12:**
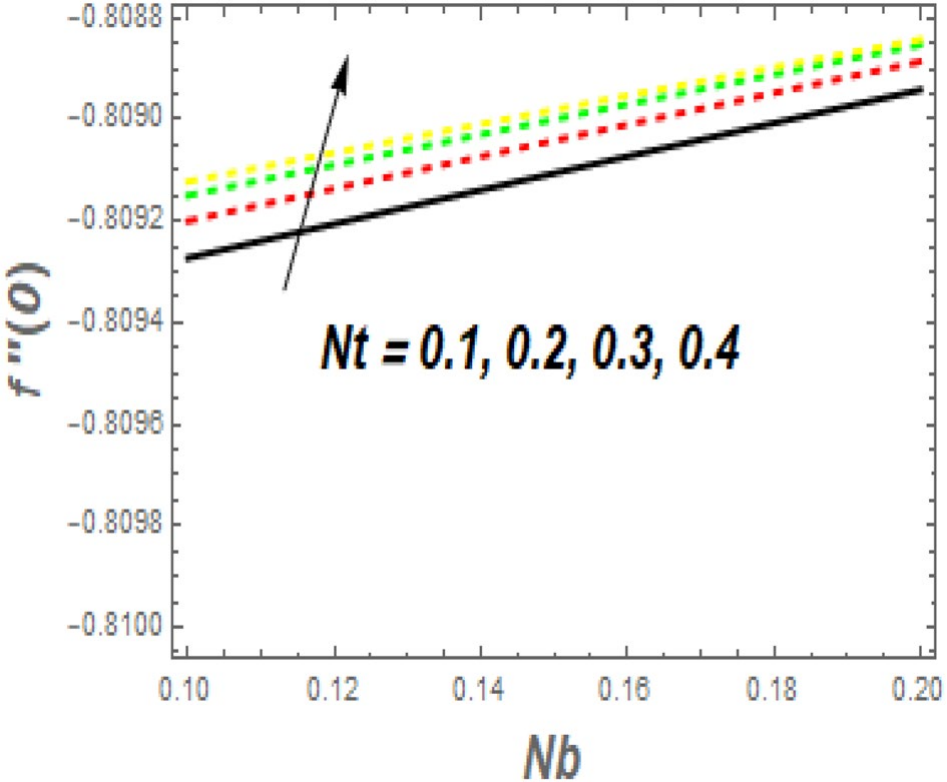
Impacts of *Nt* and $Nb$ on $C_{f} .$

**Figure 13:**
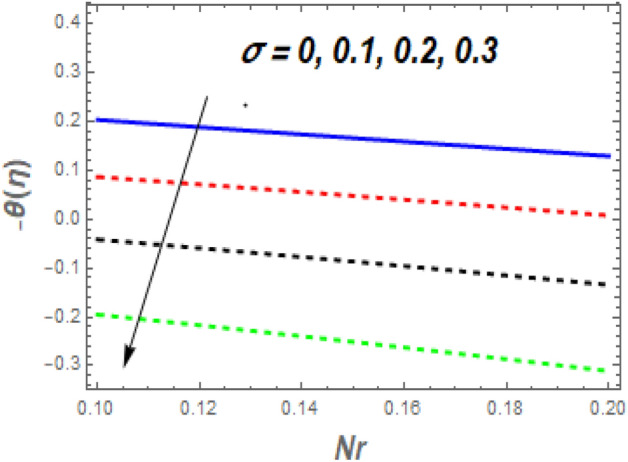
Impacts of *Nr* and $\sigma$ on $Nu_{x} .$

**Figure 14:**
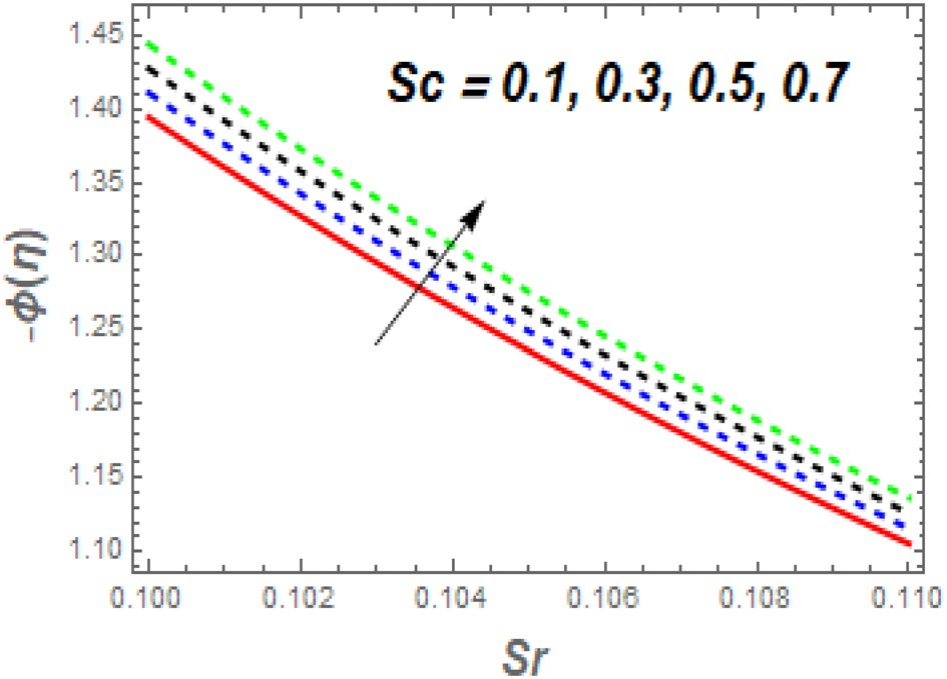
Impacts of *Sc* and $Sr$ on $Sh_{x} .$

As seen in Fig. 11, the $f^{\prime\prime}\left( 0 \right)$ growth as *M* increases while decreasing as $\alpha$ rises. Increasing the value of *M* causes a significant resistance to fluid flow owing to the Lorentz drag force, which decreases the fluid velocity and the momentum BL thickness, increasing the velocity and, as a result, the shear stress at the exponential stretching sheet. For larger values of *Nb* and *Nt*, the $f^{\prime\prime}\left( 0 \right)$ grows, as seen in Fig. 12. The reason for this is that higher values of *Nb* and *Nt* speed up the movement of nanoparticles from the surface to the free stream, causing friction between the surface and the fluid and lowering the heat transfer rate. The $- \theta^{\prime}\left( 0 \right)$ falls as the *Nr* and $\sigma$ values increase, as illustrated in Fig. 13. The reason for this is that larger values of *Nr* and $\sigma$ cause nanoparticles to move away from the surface and into the free stream, generating friction between the surface and fluid and slowing heat transmission. The fluctuations of $- \phi^{\prime}\left( 0 \right)$ with *Sc* for various amounts of *Sr* are shown in Fig. 14. According to the graph, the mass transfer rates rise as *Sc* grows, with slightly different behaviour for *Sr*. In the presence of a lower *Sc* force generated by a temperature gradient, the intensity of random movement of nanoparticles boosts their transportation rate, whereas, in the presence of a larger magnitude of *Sr* force, the transport rate declines.

## Conclusions

In this article, we have examined the time-dependent slip boundary layer flow of second-grade nanofluid over an exponentially stretching surface under the variable thermal conductivity using HAM. Also, The Soret, Dufour, and nonlinear thermal radiation impacts were all taken into account. The validity of major findings and outcomes was discussed. When the results were compared to the existing literature, they were found to be good. The impact of the relevant parameters on flow, heat, and mass transmission characteristics via a graph and briefly discussed. Therefore, some final remarks of this article are as follows.

- The magnetic and unsteady characteristics both have a decreasing impact on fluid velocity, while the second grade, Grashof and mass Grashof numbers have a significant impact on fluid velocity.
- The Prandtl number and unsteady characteristics both have a decreasing impact on the temperature, while the thermal radiation, temperature ratio, Eckert number, Brownian motion, variable thermal conductivity parameter and thermophoresis have a substantial influence on heat transfer.
- Dufour and Soret numbers have a significant impact on the quicker heat and mass transport in variable thermal conductivity compared to constant thermal conductivity respectively.
- An enhancement in the thermal conductivity parameter indicates a temperature upsurge.
- The local Nusselt number declined by growing variable conductivity and thermal radiation parameters.
- The skin friction coefficient improves with magnetic fields, Brownian motion, and thermophoresis, but diminishes when the value of a second-grade parameter increases.
- The local Sherwood number decline by growing the Soret number, but rise when the value of a Schmidt number increases.
- When compared to the assumption of constant fluid characteristics, the variable thermal conductivity is noticeably different.

## Data Availability

The data used to support the findings of this study is available from the corresponding author upon request.
